# Study on the therapeutic potential of *Dichroa febrifuga* Lour. as a novel natural anticoccidial agent for *Eimeria tenella* infection in chicks

**DOI:** 10.3389/fvets.2025.1713448

**Published:** 2026-01-02

**Authors:** Zhiting Guo, Xinrong Li, Shaobo Zhang, He Wang, Xiaoqin Luo, Yuan Liu, Xiaocheng Wei, Chengyi Li

**Affiliations:** 1 College of Pharmacy, Gansu University of Chinese Medicine, Lanzhou, China; 2 Technology Innovation Center of Traditional Chinese Veterinary, Gansu Province & Key Lab of Veterinary Pharmaceutical Development, Ministry of Agriculture and Rural Affairs, Lanzhou Institute of Husbandry and Pharmaceutical Sciences, Chinese Academy of Agricultural Sciences, Lanzhou, China

**Keywords:** anti-inflammatory, anticoccidial effects, chicks, *Dichroa febrifuga* Lour, *Eimeria tenella*

## Abstract

Avian coccidiosis, caused by the parasite *Eimeria tenella*, significantly impacts the global poultry industry. The emergence of drug resistance and concerns about chemical residues in food make the development of effective natural alternatives imperative. This study aims to evaluate the therapeutic effects of *Dichroa febrifuga* Lour. (DFL) powder on broilers infected with *E. tenella* and explore its potential mechanisms. A total of 72 sixteen-day-old white-feathered broiler chicks were randomly allocated into four groups (each with six replicates of three chicks): Control, Model, DFL, and JQCS groups. Except for the Control group, all other groups were orally administered 5 × 10^4^ *E. tenella* oocysts. The DFL and JQCS groups were fed diets containing 0.1 g/kg DFL and 15 g/kg JQCS, respectively. We established an infection model in chicks and assessed the impact of DFL on growth performance, oocyst shedding, and cecal lesion scores. Histological examination and tight junction protein expression analysis were used to assess the integrity of the cecal tissue, and RT-qPCR and ELISA were employed to quantify local and systemic inflammatory responses. In addition, we used an LPS-stimulated HD11 macrophage *in vitro* model to verify the direct anti-inflammatory effects of DFL. *In vivo*, DFL treatment significantly improved growth performance, reduced oocyst shedding, and lowered cecal lesion scores, with an excellent anticoccidial index (>170). DFL also protected the intestinal barrier integrity by upregulating the expression of ZO-1, occludin, and claudin-1. More importantly, DFL significantly alleviated the inflammatory response by inhibiting the NF-κB and MAPK signaling pathways. *In vitro*, DFL exhibited a dose-dependent effect, significantly reducing the expression of inflammatory genes at low concentrations, while high concentrations showed pro-inflammatory effects. DFL demonstrated strong therapeutic effects against avian coccidiosis by inhibiting parasite proliferation, protecting the intestinal barrier, and modulating host inflammatory responses. These findings support the potential of DFL as an effective natural alternative for controlling poultry coccidiosis and highlight the importance of dose optimization in its application.

## 1 Introduction

Avian coccidiosis is a prevalent intestinal disease in poultry caused by *Eimeria tenella* (1). With the rapid expansion of large-scale intensive poultry farming, coccidiosis has become a year-round challenge, leading to substantial economic losses in the global poultry industry. Among the nine common *Eimeria* species affecting poultry worldwide, *E. tenella* is recognized as the most pathogenic and virulent. This parasite exhibits strict host and tissue specificity, primarily infecting the crypt epithelial cells of the cecum in chickens (2, 3). During the schizogony phase of its life cycle, *E. tenella* disrupts the intestinal mucosal integrity, leading to epithelial cell degeneration, necrosis, and disintegration. Consequently, nutrient absorption is impaired, resulting in severe clinical manifestations such as bloody dysentery, weight loss, and even mortality (4). Histopathological examination of infected cecal tissues further reveals extensive structural damage, accompanied by a high density of coccidian oocysts within cecal epithelial cells. Currently, anticoccidial drugs, including polyether ionophore antibiotics and synthetic chemicals, remain the primary treatment options (5). However, the widespread use of these drugs has led to the emergence of drug-resistant *Eimeria* strains, thereby reducing their therapeutic and preventive efficacy (6–8). Furthermore, concerns regarding drug residues in animal products pose significant risks to food safety (9).

In recent years, Traditional Chinese medicine (TCM) has gained increasing attention for its potential in coccidiosis prevention and treatment. TCM offers unique advantages, including broad availability, low toxicity, minimal drug residues, and a reduced likelihood of resistance development. Additionally, TCM has been reported to enhance immunity and alleviate clinical symptoms associated with coccidiosis (10, 11). Various TCM-derived compounds have demonstrated potent anticoccidial effects. For instance, Areca nut extract not only improves the growth performance of *Eimeria*-infected chicks but also mitigates intestinal damage caused by coccidia (12). Similarly, dietary supplementation with Cinnamon bark has been shown to enhance immune function, inhibit *Eimeria* parasitism, regulate gut microbiota, and reduce intestinal inflammation (13). Furthermore, extracts from *Fructus Melia Toosendan* have been found to alleviate inflammatory responses, intestinal bleeding, and diarrhea, while simultaneously decreasing oocyst shedding and improving relative weight gain and anticoccidial efficacy (14). Other herbal extracts, such as *Artemisia sieberi, Artemisia apiacea*, and cress, have also demonstrated promising anticoccidial properties.

*Dichroa febrifuga* Lour (DFL), a medicinal herb recorded in the ancient Chinese pharmacopeia *Sheng Nong's Herbal Classic*, has been traditionally used for its expectorant and antimalarial properties (15). The primary active compound in DFL, febrifugine, has been reported to confer resistance against various *Eimeria* infections (16). Additionally, in our previous study, DFL was able to inhibit the growth and development of *E. tenella* in the intestinal mucosa, demonstrating potent anticoccidial properties, and it was also found that DFL could stimulate the host's immune cells and enhance immune function (17–19). However, the mechanism of DFL treatment for chicken coccidiosis is still unclear.

This study aimed to further evaluate the potential of DFL for treating avian coccidiosis and to preliminarily elucidate its therapeutic mechanisms. To this end, we assessed the effects of DFL on the production performance of coccidia-infected chickens and quantified its anticoccidial activity. By examining cecal gross morphology, HE staining, immunohistochemistry, and transmission electron microscopy, we evaluated DFL's ability to restore both the overall and ultrastructural integrity of the cecum in infected chicks. Furthermore, we measured inflammatory cytokine expression *in vivo* and *in vitro* to assess DFL's anti-inflammatory efficacy in *E. tenella*–infected chicks. This study aims to provide a theoretical basis for the scientific application of DFL in poultry coccidiosis treatment and to offer a novel strategy for reducing drug resistance and replacing chemical anticoccidials.

## 2 Materials and methods

### 2.1 Drugs and reagents

The DFL powder pilot products (100 g/bag, lot No. 2021111501, 1 g of DFL equivalent to 10 g of DFL crude drug), were purchased from Shijiazhuang Zhengdao Animal Pharmaceutical Co., LTD (Shijiazhuang, Hebei Province, China). The production process of DFL powder is as follows: the roots of the DFL medicinal herb are crushed, and the active components are extracted using ultrasonic-assisted extraction. The extract is then dried and pulverized, after which an appropriate amount of starch is added and mixed thoroughly (the mass ratio of DFL to starch is 9:1).

The positive control drug, Jiqiuchong San (JQCS, 500 g/bag, lot No. 220301), were purchased from AnqingKekuang Animal Pharmaceutical Co., LTD, composed of *Artemisia annua* L. (3,000 g), *Agrimonia pilosa* Ledeb (500 g), *Pleuropterus multiflorus* (Thunb.) Nakai (500 g), *Pulsatilla chinensis* (Bunge) Regel (300 g) and *Cinnamomum cassia* (L.) D. Don (260 g). The production process of JQCS involved weighing the formula components, pulverizing, sieving, and mixing them thoroughly.

The reference standards for active-ingredient identification, Febrifugine and α-Dichroine, were purchased from Sinopharm Chemical Reagent Co., Ltd. (Shanghai, China).

### 2.2 Identification of active components in DFL

DFL samples were analyzed by ultra-high-performance liquid chromatography (UHPLC; Agilent 1290, Santa Clara, CA, USA) equipped with a Kromasil C18 column (4.6 × 250 mm, 5 μm; Amsterdam, Noord-Holland, Netherlands). The mobile phase consisted of acetonitrile−0.1% phosphoric acid (10:90, v/v); detection was performed at 225 nm; column temperature was maintained at 30 °C; flow rate was 1.0 ml/min; total run time was 30 min; injection volume was 10 μl. Peak areas of the analytes were integrated and compared against those of the reference standards.

Structural characterization of Febrifugine and α-Dichroine was carried out by high-resolution electrospray ionization mass spectrometry (HRESIMS) on a Xevo G2-S QTOF (Waters, Milford, MA, USA) operated in positive-ion ESI mode. Instrument parameters were as follows: capillary voltage, 3.0 kV; source temperature, 120 °C; desolvation temperature, 350 °C; cone gas flow, 50 L/h; desolvation gas flow, 800 L/h; scan range, m/z 100–1,000; scan time, 0.2 s. Data were processed to confirm molecular formulas and key fragment ions characteristic of Febrifugine and α-Dichroine.

### 2.3 Coccidium oocysts

*E. tenella* oocysts were provided by the Laboratory of Parasitic Diseases, China Agricultural University. To ensure oocyst viability before the experiment, they were rejuvenated. The sporulation rate was assessed by microscopic examination, and only batches with a rate exceeding 85% were selected for use. The purified oocysts were then stored in a 2.5% potassium dichromate solution at 4 °C until use.

### 2.4 Animals, experimental design, and sample collection

All animal experiments were conducted in accordance with the National Institutes of Health guide for the care and use of Laboratory animals and received approval from Lanzhou Institute of Husbandry and Pharmaceutical Sciences of Chinese Academy of Agricultural Sciences (NO. 2024-009).

One-day-old white-feathered broilers were obtained from Wangmiao Poultry Farm (Jiuquan, Gansu, China). The chicks were initially housed in a SPF isolation chamber until they reached 12 days of age. Subsequently, they were transferred to a coccidiosis-free laboratory animal facility. At 14 days of age, the broilers were immunized with a Newcastle Disease-Infectious Bronchitis combined vaccine via nasal drops and reared under controlled conditions until they reached 16 days of age.

One week prior to rearing, the four walls, ventilation system, and animal cages of the experimental animal room had all undergone thorough cleaning and disinfection. The day before introducing the chicks, the ambient temperature was adjusted to 35 °C. At 3 days of age, the temperature was adjusted to 33 °C; at 7 days of age, it was reduced to 30 °C; and at 14 days of age, it was further adjusted to 28 °C, ensuring that the chicks were consistently maintained at their optimal temperature. During the experiment, each chick cage (measuring 40 × 30 × 30 cm, length × width × height) housed three chicks.

Prior to the experiment, fecal samples from the chicks were examined for three consecutive days to confirm the absence of *E. tenella* infection. Seventy-two 16-day-old chicks were then randomly assigned to four groups of six replicates each, with three chicks per replicate. The four groups were designated as Control, Model, DFL, and JQCS. The Control group was maintained on a standard diet, while the Model, DFL, and JQCS groups were each orally challenged with 5 × 10^4^ *E. tenella* oocysts. The DFL group received feed supplemented with 0.1 g/kg DFL, and the JQCS group received feed supplemented with 15 g/kg Jiqiuchong San (JQCS), both administered once daily for four consecutive days, followed by a 3-day observation period after treatment cessation. The selection of DFL dosage and the treatment cycle was determined based on our preliminary screening and drug safety tests (17, 20, 21). At a dosage of 0.1 g/kg and a treatment cycle of 4 days, DFL demonstrated favorable efficacy with no observed toxic side effects. The dosage and timing for the positive control drug JQCS were selected in accordance with the product instructions. The grouping of experimental animals is shown in Table 1.

**Table 1:** Animal grouping and administration.

**Group**	**Animal numbers**	**Subspore inoculum volume**	**Drug concentration in feed**	**Days of administration**
Control	18	—	—	—
Model	18	5 × 10^4^/chick	—	—
DFL	18	5 × 10^4^/chick	DFL (0.1 g/kg)	Combined 4 days
JQCS	18	5 × 10^4^/chick	JQCS (15 g/kg)	Combined 4 days

Control: basal diet; Model: each chick was infected with 5 × 104 Eimeria tenella oocysts; DFL: based on the Model group, supplemented with 0.1 g/kg Dichroa febrifuga Lour. powder; JQCS: based on the Model group, supplemented with 15 g/kg of the positive control drug Jiqiuchong San.

From the start of dosing, each chick's body weight and feed intake were recorded daily. These data were used to calculate the average daily feed intake (ADFI), average daily gain (ADG), and feed-to-gain ratio (F/G) for each group, providing measures of how DFL affected growth performance in *E. tenella*-infected chicks. During this process, there were two technicians involved. One technician was responsible for the *E. tenella* challenge and drug administration tasks, while the other was tasked with observation and recording (this technician was blinded to the group allocation), thereby reducing potential bias.

Before the end of the trial, blood samples were collected from the wing vein of each chick. After clotting, the samples were centrifuged at 3,000 × g for 10 min, and the serum was harvested, aliquoted, and stored at −20 °C until further use. Following blood collection, chicks were euthanized by rapid cervical disarticulation. The entire cecum was excised, photographed to document gross pathological changes, and scored. Cecal contents were then collected for oocyst counting, and cecal tissue samples were taken for H&E staining, immunohistochemistry, and transmission electron microscopy; any remaining tissue was preserved at −80 °C for RT-qPCR analysis.

### 2.5 Calculation of DFL's anticoccidial index

The anticoccidia index (ACI) is based on the formula calculated by Merck Company (22). The relative weight gain rate is based on the final body weight and the initial body weight recorded at the start of the formal trial.


$$\begin{array}{l} \text{ACI }=\;\left(\text{survival rate }+\text{ relative weight gain rate}\right)\\ -\left(\text{lesion value }+\text{ oocyst value}\right) \end{array}$$

According to ACI value, the anticoccidia effect was classified as follows: below 120, ineffective; 120–160, differential; 160–180, good; and above 180, excellent.


$$\begin{array}{l} \text{Survival rate}\\ \text{=}\left(\frac{\text{Number of surviving chicks at experiment end}}{\text{Initial number of chicks}}\right)\\ \times \text{100}\%\\ \text{Relative weight gain rate}\\ \text{=}\left(\frac{\text{mean weight gain of each treatment group}}{\text{mean weight gain of healthy control group}}\right)\\ \times \text{100}\% \end{array}$$

The score of the lesions was based on the method of Johnson and Reid (23). If the cecum lesions were not identical on both sides, the severe side was used as the standard. The scoring criteria are listed in Table 2.


$$\begin{array}{r} \text{Leison value }=\text{ the average lesion score of each experimental}\\ \text{group }\times \;10 \end{array}$$

**Table 2:** Lesion scoring criteria.

**Score**	**Standard**
0	No visible lesions were observed.
1	A few scattered petechiae were present on the cecal wall, but there was no thickening of the cecal wall, and the contents appeared normal.
2	The cecum showed more pathological changes, including significant blood presence in the cecal contents and mild thickening of the cecal wall.
3	A large amount of blood or intestinal material was found in the cecum, with blood clots, gray-white, cheese-like, banana-shaped lumps visible. The cecal wall was significantly thickened, and there was little to no fecal matter present in the cecum.
4	The cecum was filled with a large quantity of blood or intestinal material, swollen, and the intestinal contents either contained feces or none at all. Dead chicks were also assigned a score of +4.

The number of fecal oocystspergram (OPG) in the collected cecal contents was calculated using a haemocytel counting plate and converted to OPG according to the oocyst ratio (Table 3).


$$\begin{array}{r} \text{Oocyst ratio=}\left(\frac{\text{OPG of healthy group or treatment group}}{\text{OPG of infection control group}}\right)\\ \times \text{100}\% \end{array}$$

**Table 3:** Relationship between oocyst values and oocyst ratios.

**Oocyst ratio**	**0%−1%**	**1%−25%**	**26%−50%**	**51%−75%**	**>75%**
OPG	0	5	10	20	40

### 2.6 Histological analysis of cecal tissue

The collected ceca were rinsed with pre-cooled PBS buffer, blotted dry with absorbent paper, and fixed in 4% paraformaldehyde. The tissues were then dehydrated through a graded ethanol series, cleared in xylene, and embedded in paraffin. Sections were cut and stained with H&E. Histopathological changes and morphometric parameters (crypt depth, muscle layer thickness, and serosal thickness) were evaluated by light microscopy and quantified using Image Pro PLUS (Media Cybernetics, MD, USA).

For transmission electron microscopy, cecal samples were pre-fixed in 2.5% glutaraldehyde, post-fixed in 1% osmium tetroxide, and dehydrated through a graded acetone series. Samples were infiltrated sequentially with mixtures of dehydrating agent and Epon-812 resin (3:1, 1:1, 1:3) before final embedding in pure Epon-812. Semi-thin sections were first examined by light microscopy to select regions of interest in the cecal epithelium. Ultra-thin sections (60–90 nm) were then cut, stained with uranyl acetate for 10 min followed by lead citrate for 1 min at room temperature, and imaged on a JEM-1400FLASH transmission electron microscope (JEOL, Tokyo, Japan).

The immunohistochemistry protocol involves the following steps: Paraffin sections were dewaxed using an eco-friendly dewaxing agent through three sequential baths (10 min each), followed by dehydration in absolute ethanol (three changes, 5 min each) and rinsing with distilled water. Antigen retrieval was performed using a heat-mediated method (specific buffer and conditions tailored to tissue type) to prevent epitope masking, followed by cooling and PBS washing (three cycles, 5 min each). Endogenous peroxidase activity was blocked with 3% H_2_O_2_ (25 min, room temperature, dark), and nonspecific binding sites were blocked with 3% BSA or serum matching the secondary antibody host (30 min). Primary antibody (Servicebio, Wuhan, Hubei Province, China; diluted in PBS) was applied overnight at 4 °C, followed by HRP-conjugated secondary antibody (Servicebio, Wuhan, Hubei Province, China) incubation (50 min, room temperature) after PBS washes. DAB substrate was used for chromogenic detection under microscopic monitoring until brown signals appeared, terminated by rinsing. Nuclei were counterstained with hematoxylin (3 min), differentiated, and blued before dehydration through an ethanol-xylene series. Slides were mounted with resin and evaluated under a light microscope for antigen localization and expression analysis.

### 2.7 RT-qPCR analysis of inflammation-related gene expression in cecal

One cecal tissue sample per replicate was selected for RT-qPCR. Total RNA was extracted using the TaKaRa MiniBEST Universal RNA Extraction Kit (Code No. 9767; Takara Bio, Kyoto, Japan), and RNA concentrations were measured and equalized with an ultramicro UV spectrophotometer. First-strand cDNA was synthesized using the TaKaRa PrimeScript RT Reagent Kit (Code No. RR047Q; Takara Bio, Kyoto, Japan). The cDNA was serially diluted to determine the optimal concentration for downstream RT-qPCR. Quantitative PCR was performed on an ABI Q5 real-time PCR system (Thermo Fisher Scientific, Waltham, MA, USA) using TB Green Premix Ex Taq (Code No. RR420A; Takara Bio, Kyoto, Japan). *GAPDH* served as the internal reference gene, and relative expression levels were calculated by the 2^−Δ*ΔCt*^ method. Target genes, all inflammation-associated, included tumor necrosis factor-α (*TNF-*α), C-reactive protein (*CRP*), Interleukin (IL)-1β, *IL-6, IL-8*, IL-10, nuclear factor kappa-B (*NF-*κ*B*), toll-like receptor (TLR)-2, *TLR-4, P38* MAPK (*P38*), c-Jun N-terminal kinase (JNK)1, *JNK2*, transforming growth factor-β1 (*TGF*β*1*). Primer sequences are listed in Table 4.

**Table 4:** List of mRNA primers related to inflammation.

**Gene**	**Name**	**Sequence (5^′^ → 3)**	**Accession number**
*TNF-α*	F:	GAGCGTTGACTTGGCTGTC	HQ739087.1
	R:	AAGCAACAACCAGCTATGCAC	
*CRP*	F:	CCGAGTTCTGGCTCAACGG	NM_001039564
	R:	CCCCCGTGAACGAGTTGTAG	
*IL-1β*	F:	ACTGGGCATCAAGGGCTA	AJ245728
	R:	GGTAGAAGATGAAGCGGGTC	
*IL-6*	F:	AAATCCCTCCTCGCCAATCT	AJ250838
	R:	CCCTCACGCTCTTCTCCATAAA	
*IL-8*	F:	ATGAACGGCAAGCTTGGAGCTG	HQ739083.1
	R:	TCCAAGCACCTCTCTTCCATCC	
*IL-10*	F:	GCTGAGGGTGAAGTTTGAG	NM_001004414
	R:	TGATGACTGGTGCTGGTCT	
*NF-κB*	F:	TCAATGTGCCAATGGAGGAG	D13721.1
	R:	TCAATGTGCCAATGGAGGAG	
*TLR-2*	F:	ACCTGGCCCATAACAGGATA	AB046119
	R:	ATGGAGCTGATTTGGTTGGA	
*TLR-4*	F:	GTTTGACATTGCTCGGTCCT	NM_001030693.2
	R:	CCTCCTCAGATATCGGGACA	
*P38*	F:	TGTGTTCACCCCTGCCAAGT	XM_001232615
	R:	GCCCCCGAAGAATCTGGTAT	
*JNK1*	F:	GGGTGCATTATGGGCGAAAT	XM_004942132
	R:	TTCTGGGCACGGTGTTCCTA	
*JNK2*	F:	AGCAGCCTCGATGCCTTGAC	XM_046900303
	R:	CAAGCAATTCAGGCCCAATG	
*TGFβ1*	F:	CGGGACGGATGAGAAGAA	M31160
	R:	TCGGCGCTCCAGATGTAC	
*GAPDH*	F:	TGCTGCCCAGAACATCATCC	NM_204305.1
	R:	ACGGCAGGTCAGGTCAACAA	

TNF-α, tumor necrosis factor-α; CRP, C-reactive protein; IL, interleukin; NF-κB, nuclear factor kappa-B; TLR, toll-like receptor; P38, P38 MAPK; JNK, c-Jun N-terminal kinase; TGFβ1, transforming growth factor-β1; GAPDH, glyceraldehyde-3-phosphate dehydrogenase.

### 2.8 ELISA measurement of inflammatory proteins in serum

To evaluate the improvement effect of drugs on systemic inflammation, we used ELISA to detect the content of inflammatory proteins in serum, including TNF-α (Cat. No. ml002790), IL-1β (Cat. No. ml059835A), CRP (Cat. No. ml299636V), IL-6 (Cat. No. ml059839), IL-8 (Cat. No. ml1059840), IL-10 (Cat. No. ml059830), p38 (Cat. No. ml952141v), JNK1 (Cat. No. YJ624058), JNK2 (Cat. No. YJ624096), and TGF-β1 (Cat. No. ml002780A). All ELISA kits were purchased from the Shanghai Enzyme-linked Biotechnology Co., Ltd. (Shanghai, China), following the manufacturers' protocols.

### 2.9 Cellular validation experiments

Chicken macrophage HD11 cells, maintained in our laboratory, were cultured in high-glucose DMEM (Thermo Fisher Scientific) supplemented with 10% fetal bovine serum (GIBCO; Thermo Fisher Scientific) at 37 °C with 5% CO_2_. When cells reached ~85% confluence, the Model and DFL treatment groups were stimulated with 1 μg/ml LPS (Solarbio, Beijing, China) for 9 h to induce inflammation. Afterward, DFL was added at 1, 5, 10, or 20 μg/ml for 16 h. Six groups were tested in total—Control, LPS alone, and four DFL dose groups—with five replicates per group. At the end of treatment, total RNA was extracted for RT-qPCR of inflammatory markers (*TNF-*α, *IL-1*β, *IL-6, IL-8, IL-10, NF-*κ*B, TLR-2*, and *TLR-4*) using the same reagents and methods as described above.

### 2.10 Statistical analysis

In the animal trial, each chick served as the experimental unit; in the cell assay, each well was the experimental unit. A total of 18 chicks per group were used to analyze growth performance and anticoccidial index. For RT-qPCR and ELISA, six samples per group were randomly selected (one per replicate). Data were analyzed with SPSS 26.0 (Chicago, IL, USA). One-way ANOVA followed by Tukey's *post hoc* test was used to compare means among groups. Results are expressed as mean ± standard deviation (SD), and *P* < 0.05 was considered statistically significant.

## 3 Results

### 3.1 Identification of bioactive components in DFL

**Figure 1:**
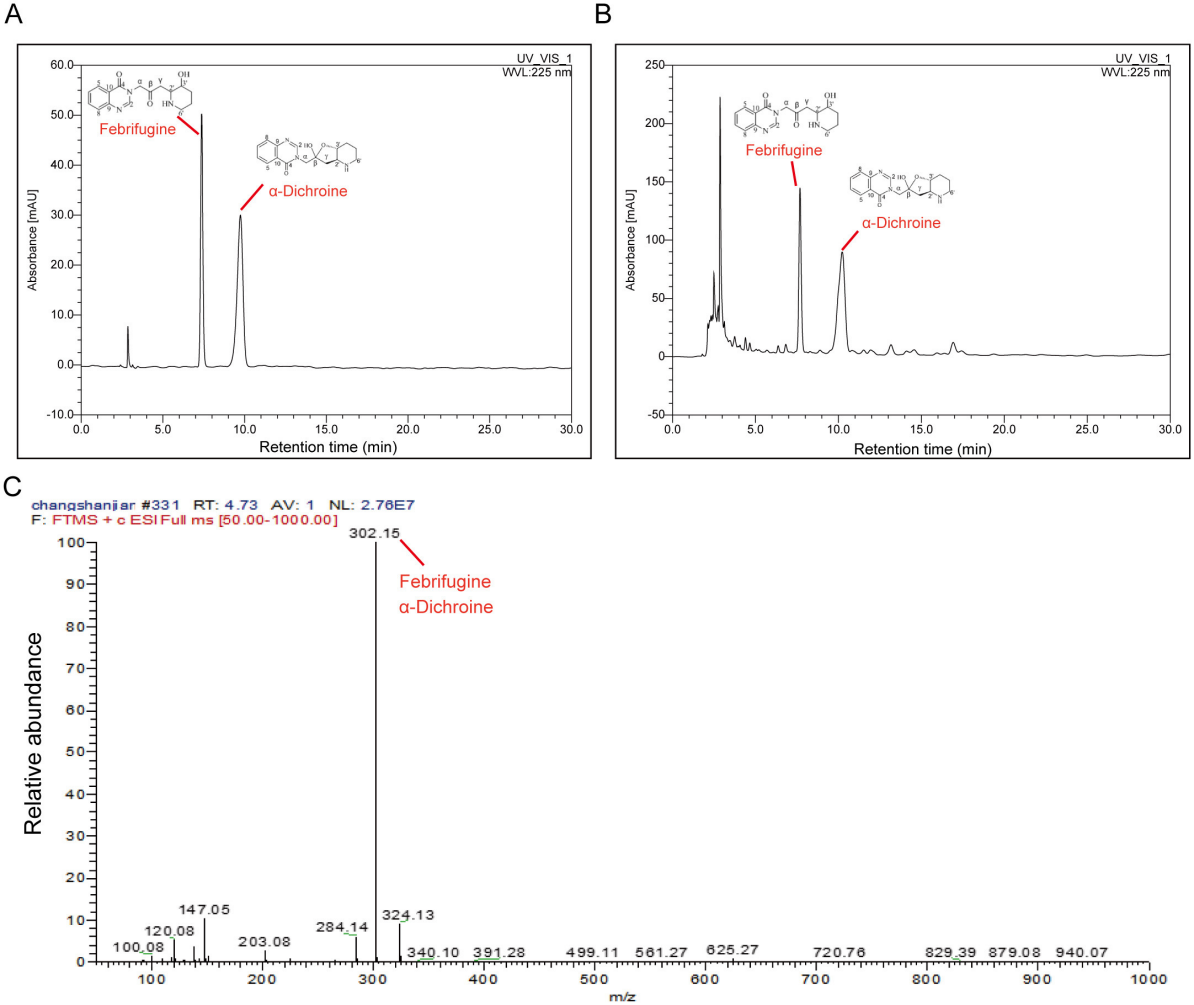
Identification and quantification of the active components in *Dichroa febrifuga Lour*. powder. **(A)** High-performance liquid chromatography chromatogram of the reference standards. **(B)** Identification and quantification of active components in a *Dichroa febrifuga Lour*. powder sample. **(C)** High-resolution electrospray ionization mass spectrometry spectrum.

Using HPLC analysis, we identified two bioactive compounds in DFL, both of which produced well-resolved chromatographic peaks. These compounds were Febrifugine and α-Dichroine (Figures 1A, B). The quantified content of febrifugine was 1.924 mg/g, and that of α-dichroine was 5.256 mg/g. To further confirm their structures, we subjected them to high-resolution electrospray ionization mass spectrometry (HRESIMS). As shown in Figure 1C, both substances are constitutional isomers, sharing the same molecular formula (C16H19O3N3) and identical mass spectra.

### 3.2 The effects of DFL on growth performance of *E. tenella*-infected chicks

*E. tenella* infection severely impaired chick growth performance, so we measured standard performance parameters to evaluate DFL's impact (Table 5). At the start of the trial, average initial weights did not differ among groups. By the end, the Model group (infected untreated) had a significantly lower average final weight than the Control group (*P* < 0.05). Chicks in both the DFL-treated and JQCS (positive control) groups weighed significantly more than those in the Model group (*P* < 0.05), with no significant difference between DFL and JQCS. *E. tenella* infection also reduced average daily gain (ADG) in the Model group compared to Control (*P* < 0.05), whereas both DFL and JQCS treatments restored ADG to levels significantly higher than the Model (*P* < 0.05). The Model group showed an elevated ADFI relative to Control, which was normalized by DFL. F/G was higher in the Model group vs. Control but was significantly reduced by DFL and JQCS. These data demonstrate that DFL effectively improves growth performance in *E. tenella*-infected chicks.

**Table 5:** Effects of DFL powder on growth performance in coccidia-infected chicks.

**Group**	**Average initial weight (g) 16**	**Average final weight (g) 21**	**ADG (g)**	**ADFI (g)**	**F/G**
Control	245.35 ± 5.78	484.28 ± 7.12^a^	34.13 ± 7.12^a^	50.48	1.48
Model	242.46 ± 7.90	442.87 ± 3.50^b^	28.63 ± 3.50^b^	55.32	1.93
DFL	243.06 ± 8.14	463.76 ± 3.23^c^	31.53 ± 3.23^c^	51.23	1.62
JQCS	245.94 ± 6.53	454.61 ± 11.95^c^	29.81 ± 1.95^c^	49.09	1.65

Different letters in the same column mean significant difference between treatment groups (*P* < 0.05), same letter in the same column mean no significant difference between treatment groups (P > 0.05).

Control: basal diet; Model: each chick was infected with 5 × 104 Eimeria tenella oocysts; DFL: based on the Model group, supplemented with 0.1 g/kg Dichroa febrifuga Lour. powder; JQCS: based on the Model group, supplemented with 15 g/kg of the positive control drug Jiqiuchong San.

ADG, average daily weight gain; ADFI, the average daily feed intake; F/G, feed-to-gain ratio.

### 3.3 Anticoccidial activity of DFL

To elucidate the mechanism underlying the improved growth performance observed in DFL-treated, coccidia-infected chicks, we quantified the oocyst output and evaluated cecal lesions following DFL administration. The results indicated that no oocysts were detected in Control group, whereas Model group exhibited a substantial oocyst load. Notably, oocyst counts in DFL group and JQCS group were significantly reduced (Table 6). Cecal lesion scores, another important anticoccidial indicator, were markedly higher in the Model group compared to Control (*P* < 0.05) and were significantly reduced by DFL and JQCS treatment (Table 7, *P* < 0.05).

**Table 6:** Effects of DFL powder on OPG in coccidia-infected chicks.

**Group**	**OPG ( × 10^6^)**	**Oocyst ratio (%)**	**Oocyst score**
Control	0	0	0
Model	10.58	100	40
DFL	3.67	34.69	10
JQCS	4.63	43.76	10

Control: basal diet; Model: each chick was infected with 5 × 104 Eimeria tenella oocysts; DFL: based on the Model group, supplemented with 0.1 g/kg Dichroa febrifuga Lour. powder; JQCS: based on the Model group, supplemented with 15 g/kg of the positive control drug Jiqiuchong San.

OPG, oocysts per gram.

**Table 7:** Effects of DFL powder on cecal lesion scores in coccidia-infected chicks.

**Group**	**Cecal lesion score**	**Lesion value**
Control	0.00^a^	0
Model	2.10 ± 0.74^b^	21
DFL	0.51 ± 0.66^c^	5.1
JQCS	0.85 ± 0.81^c^	8.5

Different letters in the same column mean significant difference between treatment groups (*P* < 0.05), same letter in the same column mean no significant difference between treatment groups (*P* > 0.05).

Control: basal diet; Model: each chick was infected with 5 × 104 Eimeria tenella oocysts; DFL: based on the Model group, supplemented with 0.1 g/kg Dichroa febrifuga Lour. powder; JQCS: based on the Model group, supplemented with 15 g/kg of the positive control drug Jiqiuchong San.

The ACI, calculated via the Merck formula, integrates survival rate, weight gain, lesion score, and oocyst output. Except for the Model group, all groups achieved 100% survival (Table 8). The Model group's relative weight gain rate was depressed by infection but was restored most effectively by DFL and to a similar extent by JQCS. Consequently, both DFL and JQCS achieved ACI values above 170, indicating strong anticoccidial efficacy, with DFL showing superior therapeutic performance (Table 8).

**Table 8:** The anticoccidia index (ACI) comparison between experimental groups.

**Group**	**Survival rate (%)**	**Relative weight gain rate (%)**	**Cecal lesion value**	**Oocyst score**	**ACI**
Control	100.00	100.00	0	0	200.00
Model	88.89	86.32	21.00	40	115.32
DFL	100.00	95.06	5.10	10	180.00
JQCS	100.00	89.88	8.50	10	171.38

Control: basal diet; Model: each chick was infected with 5 × 104 Eimeria tenella oocysts; DFL: based on the Model group, supplemented with 0.1 g/kg Dichroa febrifuga Lour. powder; JQCS: based on the Model group, supplemented with 15 g/kg of the positive control drug Jiqiuchong San.

### 3.4 DFL alleviates cecal tissue damage

*E. tenella* specifically invades the cecum of chicks, causing substantial structural damage. To assess the protective effect of DFL on cecal tissue architecture, we conducted histopathological evaluations including H&E staining, and transmission electron microscopy.

**Figure 2:**
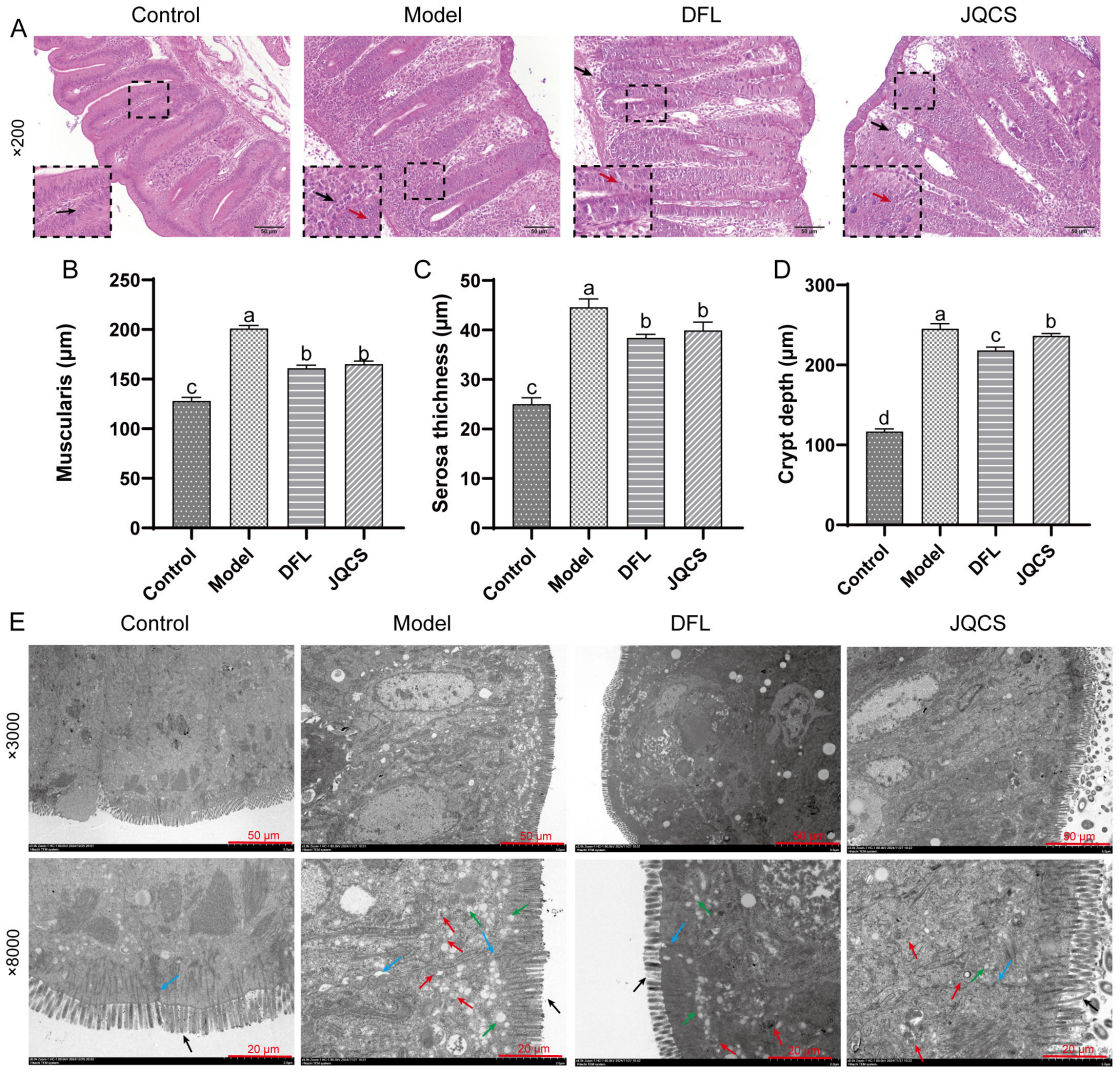
Histopathological analysis of chick cecum. **(A)** H&E staining of chick cecum. The scale bar represents a length of 50 μm. **(B)** Changes in muscularis thickness; **(C)** Changes in serosa thickness; **(D)** Changes in crypt depth. **(E)** Transmission electron microscopy (TEM) image of cecal tissue. The black arrows represent the microvilli of the intestinal epithelial cells, the blue arrows represent the tight junctions between cells, the red arrows represent the merozoites of *E. tenella*, and the green arrows represent the vacuoles formed by merogony expansion of the coccidian parasite. *n* = 6, data are presented as mean ± SD. Different letters (a–c) indicate significant differences between groups (*P* < 0.05). Control: basal diet; Model: each chick was infected with 5 × 104 *Eimeria tenella* oocysts; DFL: based on the Model group, supplemented with 0.1 g/kg *Dichroa febrifuga Lour*. powder; JQCS: based on the Model group, supplemented with 15 g/kg of the positive control drug Jiqiuchong San.

In the Control group, the cecal glands were intact, exhibiting normal histological architecture without signs of necrosis, and goblet cells along with other epithelial cells appeared healthy (Figure 2A). In the Model group, the glandular structure was obscured, crypts were disrupted, neutrophilic infiltration was evident in the epithelium and lumen, submucosal inflammation was pronounced, and numerous coccidian gametophytes had invaded the tissue. In the DFL group, cecal glands were largely preserved, with only mild pathological alterations and minimal goblet cell damage. The JQCS group showed the presence of coccidian gametophytes within the glands and epithelial cells, and a small number of inflammatory cells were noted at the base of the crypts.

Morphometric parameters such as cecal muscle layer thickness, serosal membrane thickness, and crypt depth serve as indicators of tissue integrity. These measurements were significantly lower in the Control group compared to the other three groups (*P* < 0.05), while the Model group showed significantly elevated values relative to the DFL and JQCS groups (Figures 2B, C, *P* < 0.05). Notably, the DFL group exhibited significantly reduced muscle layer thickness and crypt depth compared to the JQCS group (*P* < 0.05), indicating a more potent protective effect of DFL on cecal tissue structure (Figures 2C, D).

The TEM results primarily focused on the integrity of the intestinal epithelial cell structure and the presence of merozoites within the cells (Figure 2E). In the Control group, the tight junctions between cecal epithelial cells remained intact, the microvilli of the intestinal epithelial cells were neatly arranged, and no *E. tenella* merozoites were observed. In the Model group, the microvilli were dense and orderly arranged, showing no immediate damage from *E. tenella*. However, the tight junctions between cells were opened, leading to impaired barrier function. A large number of *E. tenella* merozoites were observed within the cells, along with numerous intracellular vacuoles resulting from *E. tenella* merogony. *E. tenella* caused relatively severe damage to the cells. In the DFL group, the microvilli were densely and neatly arranged, the tight junctions between cells remained intact, and a small number of merozoites and vacuoles were observed. This indicates that DFL effectively kills *E. tenella*. In the JQCS group, the microvilli were sparse and loosely arranged, and the tight junctions between cells were slightly opened. A small number of merozoites and vacuoles were observed within the cells. Overall, these results suggest that DFL effectively alleviates cecal tissue damage induced by *E. tenella* infection and helps maintain intestinal barrier stability.

### 3.5 DFL enhances tight junction protein expression in cecal tissue

**Figure 3:**
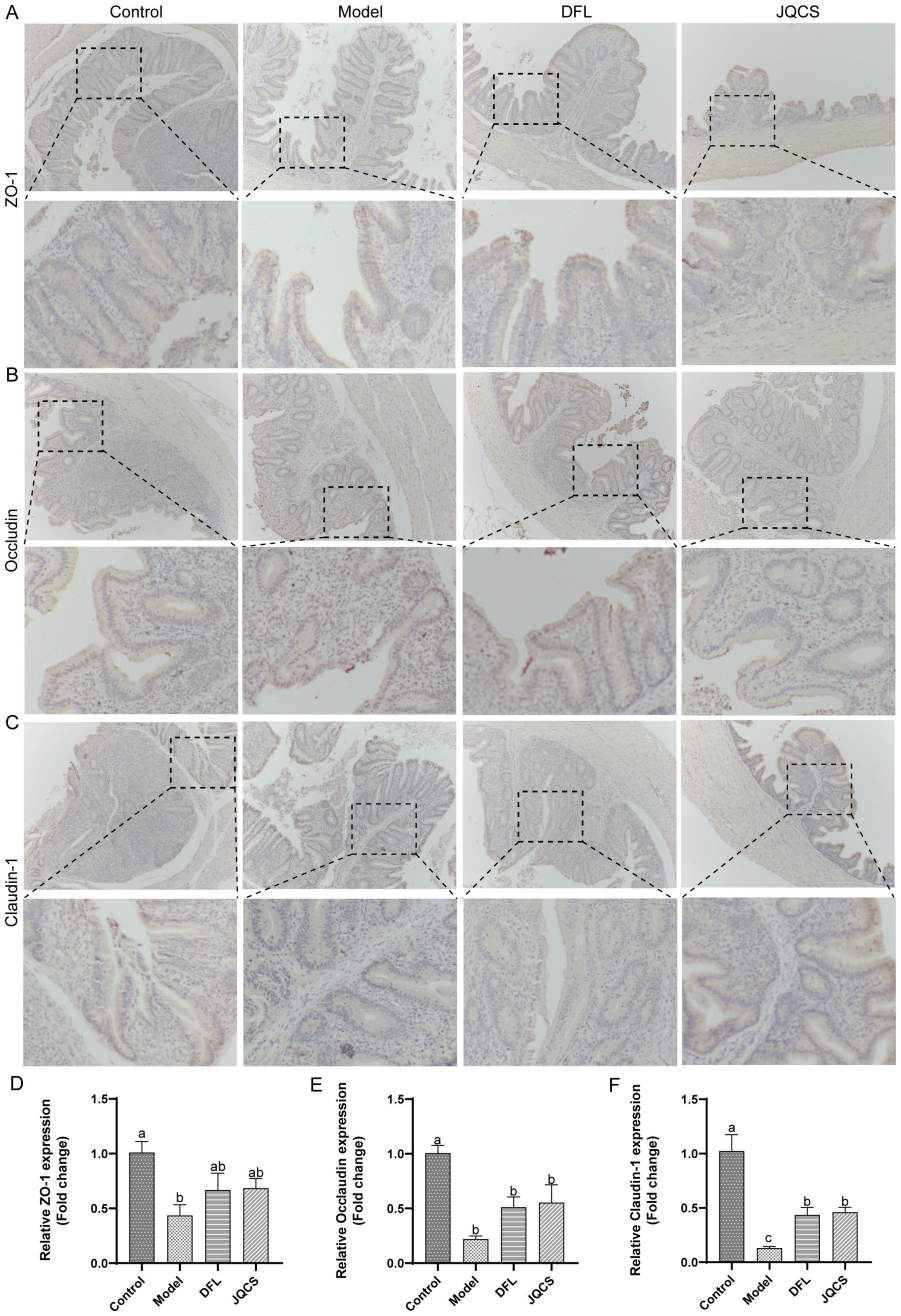
Analysis of tight junction protein and gene expression in the chick cecum. **(A)** Expression of ZO-1 protein. **(B)** Expression of Occludin protein. **(C)** Expression of Claudin-1 protein. **(D)** Gene expression of ZO-1. **(E)** Gene expression of Occludin. **(F)** Gene expression of Claudin-1. *n* = 6, data are presented as mean ± SD. Different letters (a–c) indicate significant differences between groups (*P* < 0.05). Control: basal diet; Model: each chick was infected with 5 × 104 *Eimeria tenella* oocysts; DFL: based on the Model group, supplemented with 0.1 g/kg *Dichroa febrifuga Lour*. powder; JQCS: based on the Model group, supplemented with 15 g/kg of the positive control drug Jiqiuchong San.

Tight junction proteins are critical for maintaining the integrity of the intestinal physical barrier. Immunohistochemical analysis revealed strong expression of ZO-1, Occludin, and Claudin-1 in the Control group (Figures 3A–C). In contrast, the Model group exhibited reduced expression of these proteins, indicating disruption of the cecal epithelial barrier due to *E. tenella* infection. Following DFL or JQCS treatment, the expression levels of ZO-1, Occludin, and Claudin-1 were markedly increased compared to the Model group.

This upregulation was further confirmed by RT-qPCR. As shown in Figures 3D–F, mRNA expression levels of ZO-1, Occludin, and Claudin-1 were significantly lower in the Model group than in the Control group (*P* < 0.05), while DFL treatment significantly elevated their expression (*P* < 0.05). These findings indicate that DFL enhances the expression of tight junction proteins and thereby contributes to the maintenance of intestinal epithelial barrier integrity.

### 3.6 DFL attenuates cecal and systemic inflammation

*E. tenella* infection causes severe destruction of cecal tissue and triggers intense inflammatory responses, which can further exacerbate intestinal structural damage. To assess the anti-inflammatory effects of DFL, we examined the mRNA expression of inflammation-related genes in cecal tissue and measured the levels of inflammation-related proteins in serum.

**Figure 4:**
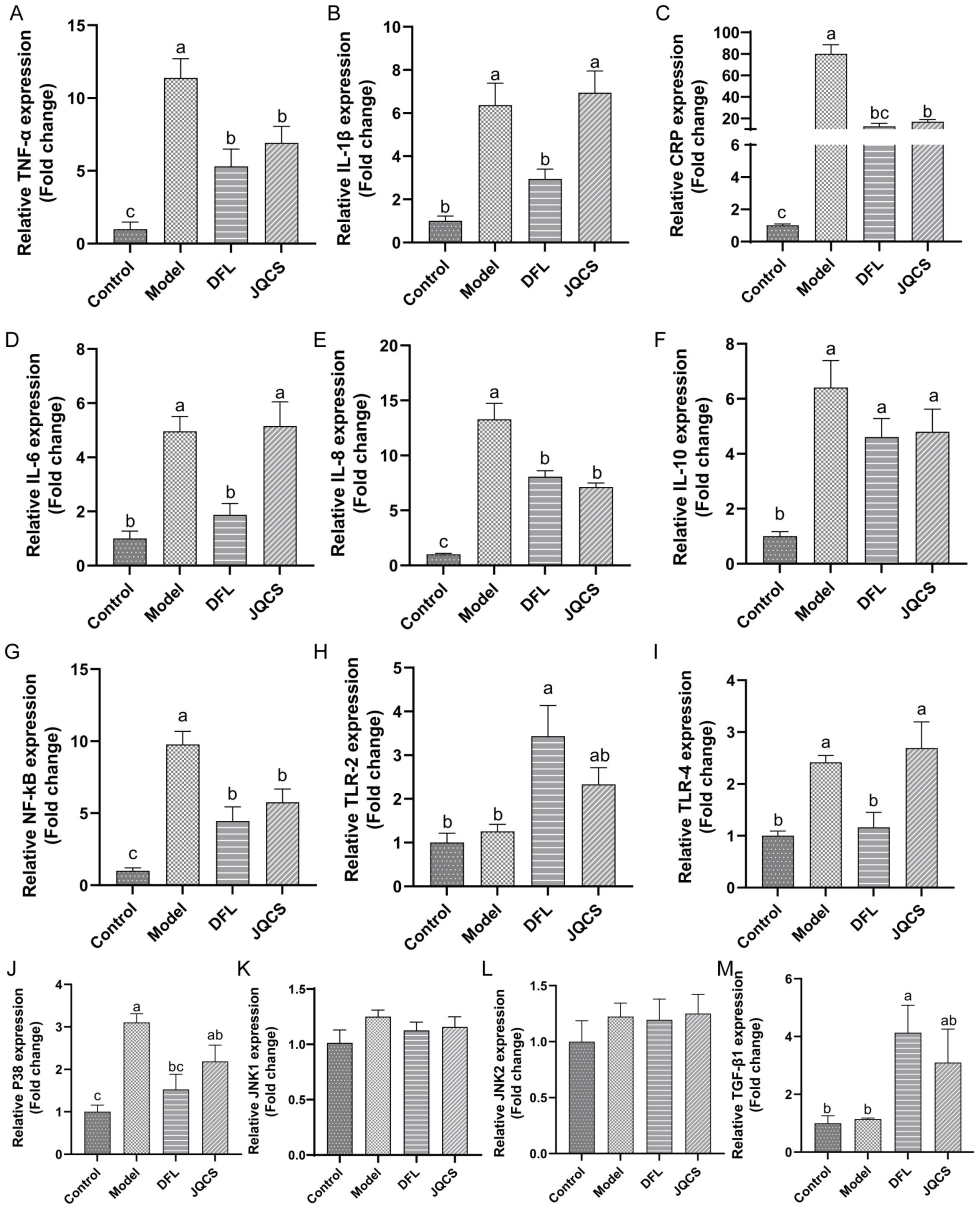
Ameliorative effects of DFL powder on cecal inflammation. **(A–F)** Gene expression of inflammation-related cytokines. **(G–I)** Gene expression related to the NF-κB signaling pathway. **(J–M)** Gene expression related to the MAPK signaling pathway. *n* = 6, data are presented as mean ± SD. Different letters (a–c) indicate significant differences between groups (*P* < 0.05). TNF-α, tumor necrosis factor-α; CRP, C-reactive protein; IL, interleukin; NF-κB, nuclear factor kappa-B; TLR, toll-like receptor; P38, P38 MAPK; JNK, c-Jun N-terminal kinase; TGFβ1, transforming growth factor-β1. Control: basal diet; Model: each chick was infected with 5 × 104 *Eimeria tenella* oocysts; DFL: based on the Model group, supplemented with 0.1 g/kg *Dichroa febrifuga Lour*. powder; JQCS: based on the Model group, supplemented with 15 g/kg of the positive control drug Jiqiuchong San.

As shown in Figure 4, the mRNA expression levels of inflammatory markers *TNF-*α, *IL-1*β, *CRP*, and *IL-6* in the ceca of the Model group were significantly higher than those in the Control group (Figures 4A–D, *P* < 0.05). Additionally, the mRNA levels of *IL-8* and *IL-10* were also significantly elevated in the Model group (Figures 4E, F), indicating pronounced local inflammation in the cecal tissue.

We further evaluated the expression of genes involved in the *NF-*κ*B* signaling pathway and its upstream TLRs. The results demonstrated that *E. tenella* infection significantly activated the *NF-*κ*B* pathway and upregulated *TLR-4* mRNA expression (Figures 4G–I, *P* < 0.05). Moreover, the expression of *p38*, a key component of the MAPK signaling pathway, was markedly increased in response to infection (*P* < 0.05). However, the mRNA expression of *JNK1, JNK*2, and *TGF-*β*1* was not significantly affected by *E. tenella* (Figures 4K–M).

Treatment with DFL and the positive control drug JQCS reversed these changes. DFL significantly reduced the mRNA expression levels of inflammatory markers *TNF-*α, *CRP, IL-6*, and *IL-8* (Figures 4A, C, D, *P* < 0.05). In addition, DFL also significantly downregulated the expression of *NF-*κ*B* and *TLR-4* (Figures 4G, I, *P* < 0.05), and suppressed the expression of *p38* (*P* < 0.05) while significantly upregulating *TGF-*β*1* (Figures 4J, M, *P* < 0.05). No significant effect was observed on *JNK1* and *JNK2* expression levels (Figures 4K, L). Notably, *TLR-4* expression in the JQCS group did not differ significantly from that in the Model group, while DFL significantly suppressed its expression (*P* < 0.05). For the remaining inflammatory genes, the effects of DFL were comparable to those of JQCS, suggesting that DFL's anti-inflammatory activity is similar to or potentially superior to that of JQCS.

**Figure 5:**
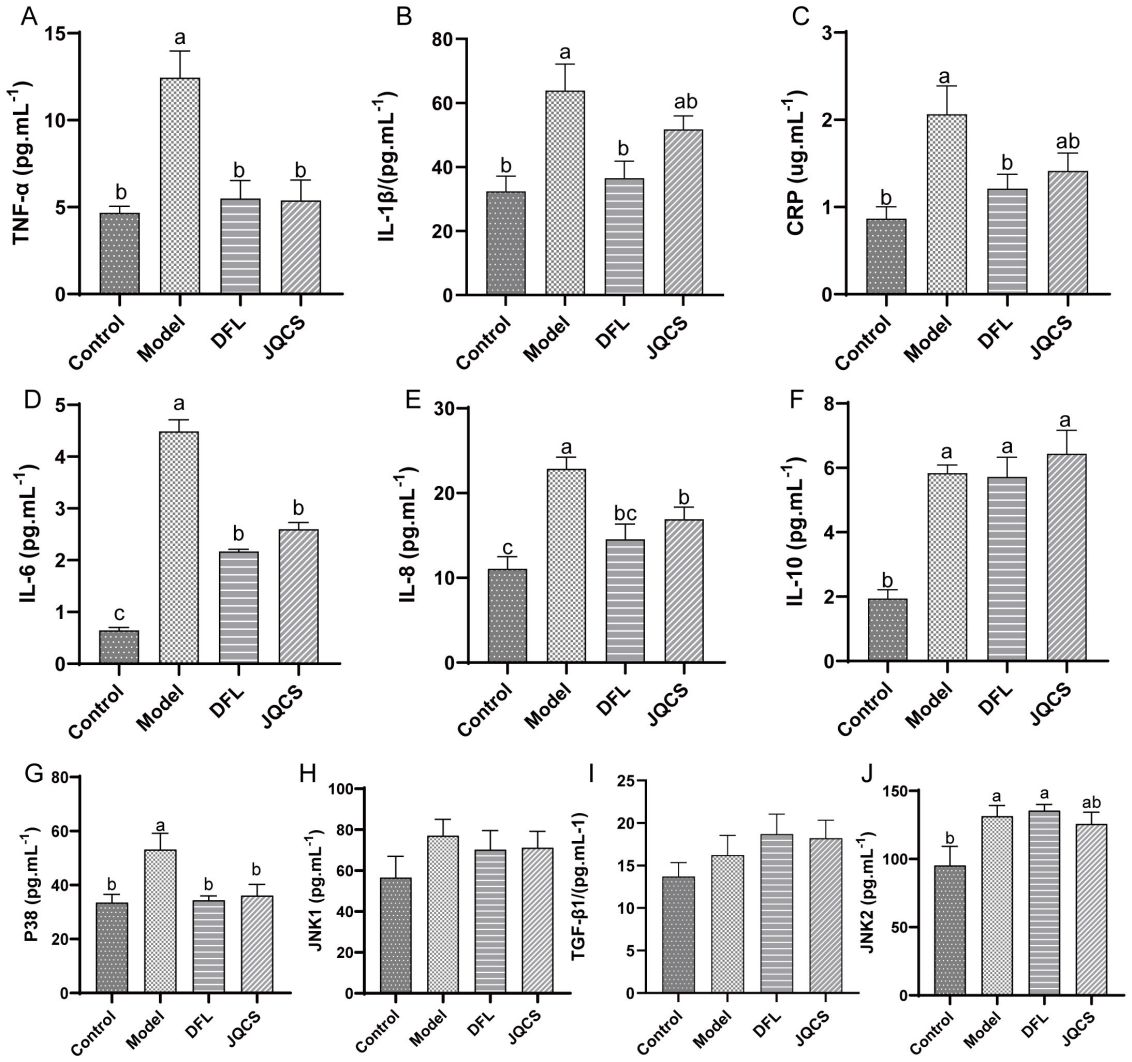
Effects of DFL powder on systemic inflammation. **(A–F)** Changes in levels of inflammation-related cytokines. **(G–J)** Protein expression related to the MAPK signaling pathway. *n* = 6, data are presented as mean ± SD. Different letters (a–c) indicate significant differences between groups (*P* < 0.05). Control: basal diet; Model: each chick was infected with 5 × 104 *Eimeria tenella* oocysts; DFL: based on the Model group, supplemented with 0.1 g/kg *Dichroa febrifuga Lour*. powder; JQCS: based on the Model group, supplemented with 15 g/kg of the positive control drug Jiqiuchong San. TNF-α, tumor necrosis factor-α; CRP, C-reactive protein; IL, interleukin; P38, P38 MAPK; JNK, c-Jun N-terminal kinase; TGFβ1, transforming growth factor-β1.

While RT-qPCR provides insight into gene expression, protein levels more directly reflect disease phenotypes. Therefore, we used ELISA to assess the concentrations of inflammatory proteins in serum. As shown in Figure 5, *E. tenella* infection significantly increased serum levels of TNF-α, IL-1β, CRP, IL-6, IL-8, and IL-10 (Figures 5A–F, *P* < 0.05). DFL treatment significantly reduced the levels of TNF-α, IL-6, and IL-8 (*P* < 0.05). JQCS treatment also significantly decreased the levels of TNF-α, IL-6, and IL-8 (Figures 5A, D, E, *P* < 0.05). *E. tenella* also elevated protein levels of p38 and JNK2 in the MAPK signaling pathway (*P* < 0.05), while both DFL and JQCS treatments significantly reduced p38 protein levels (*P* < 0.05). These results indicate that DFL exerts a potent inhibitory effect on *E. tenella*-induced inflammation at the protein level.

### 3.7 *In vitro* validation of the anti-inflammatory effects of DFL

To confirm whether the anti-inflammatory effects of DFL were due to direct action, we employed an LPS-induced inflammation model in HD11 cells and used RT-qPCR to assess the expression of inflammation-related genes.

**Figure 6:**
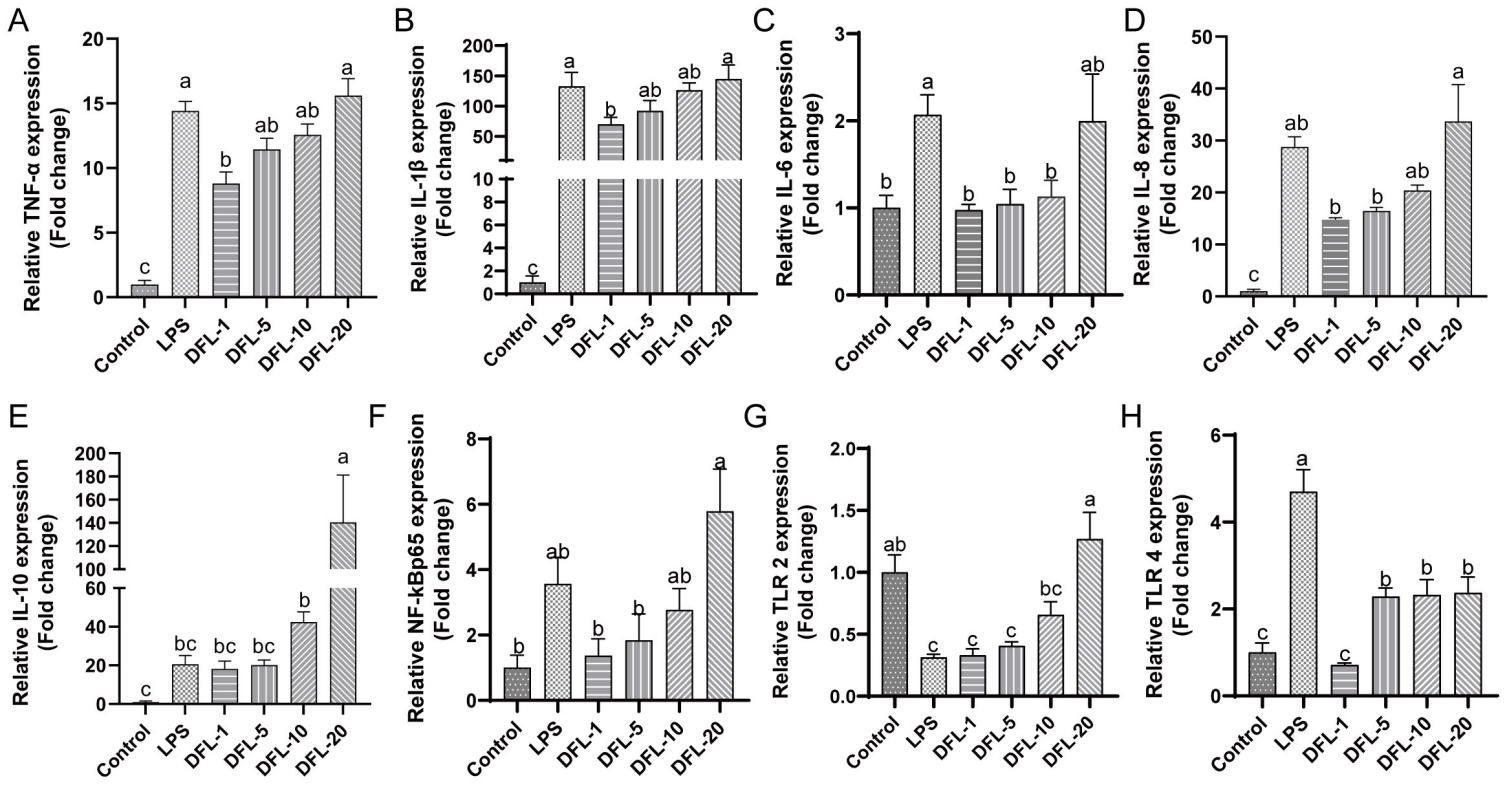
Effects of DFL powder on an LPS-induced cellular inflammation model. **(A–E)** Expression of inflammation-related genes. **(F–H)** Expression of genes related to the NF-κB signaling pathway. *n* = 5, data are presented as mean ± SD. Different letters (a–c) indicate significant differences between groups (*P* < 0.05). Control: basal diet; Model: each chick was infected with 5 × 104 *Eimeria tenella* oocysts; DFL: based on the Model group, supplemented with 0.1 g/kg *Dichroa febrifuga Lour*. powder; JQCS: based on the Model group, supplemented with 15 g/kg of the positive control drug Jiqiuchong San. TNF-α, tumor necrosis factor-α; CRP, C-reactive protein; IL, interleukin; NF-κB, nuclear factor kappa-B; TLR, toll-like receptor.

As shown in Figure 6, LPS significantly upregulated the expression of pro-inflammatory genes *TNF-*α, *IL-1*β, and *IL-6* (*P* < 0.05), successfully inducing an inflammatory response in HD11 cells. Low-dose DFL (1 μg/ml) effectively attenuated this response, significantly reducing the gene expression of *TNF-*α, *IL-1*β, *IL-6, IL-8, NF-*κ*B*, and *TLR-4* compared to the LPS group (*P* < 0.05).

However, high-dose DFL (20 μg/ml) had the opposite effect, significantly increasing the expression of *TNF-*α, *IL-1*β, *IL-6, IL-8, IL-10, NF-*κ*B, TLR-2*, and *TLR-4* (*P* < 0.05) compared to the low-dose DFL group. These findings suggest that while low doses of DFL exert anti-inflammatory effects, high doses may promote inflammation.

## 4 Discussion

Avian coccidiosis is a complex intestinal parasitic disease caused by *Eimeria* protozoa (2, 24). *E. tenella* specifically parasitizes the epithelial cells of the chick cecum, leading to destruction of intestinal tissue and inflammation (25). These pathological changes result in fever, reduced feed intake, impaired nutrient absorption, and increased susceptibility to secondary bacterial infections (26). According to evidence-based principles in TCM, treatment strategies primarily aim to clear heat and dampness and cool the blood to stop dysentery. DFL, a well-known TCM herb, has traditionally been used for its antimalarial, antipyretic, and expectorant properties (27). Our experimental results demonstrate that DFL exhibits significant anticoccidial activity against *E. tenella* infection. The OPG in the DFL-treated group were significantly lower than those in the model group, and the cecal lesion scores were also notably reduced, indicating effective control of the severity of coccidial infection. The ACI, a key metric for evaluating anticoccidial efficacy, exceeded 170 in the DFL-treated group, suggesting a “good” to “excellent” level of efficacy—surpassing even the positive control group treated with JQCS. This result is consistent with the antiparasitic properties of the active components in DFL, such as febrifugine and α-Dichroine (27). Compared with other studies, such as Ma et al. (28) using *Areca catechu* L. extract powder to treat *E. tenella*, this drug can also effectively reduce OPG, increase ACI, and exhibit significant inhibitory effects against *E. tenella*. However, this drug requires a higher dosage (0.25 g/kg of feed) and a 10-day treatment course, which increases both economic costs and labor intensity in farming practices. In contrast, DFL only requires a lower dosage (0.1 g/kg of feed) and a shorter treatment duration (4 days) to effectively inhibit *E. tenella* (29). The dosage and application duration of DFL have undergone extensive experimental validation (20) and have passed Phase I, II, and III clinical trials for new veterinary drug approval. Previous studies have also confirmed that DFL exhibits the best therapeutic efficacy during the early stage of coccidian infection (30). The study further confirms that DFL is not only effective against *Plasmodium* species but also shows significant therapeutic potential against avian coccidiosis. These findings provide experimental support for the development of DFL as a novel anticoccidial agent.

*E. tenella* infection damages the intestinal barrier and hinders nutrient absorption, ultimately impairing growth performance (31, 32). In our study, we similarly found that *E. tenella* significantly suppressed the growth performance of chicks, as evidenced by decreased ADG, increased F/G, and reduced body weight. However, DFL treatment effectively reversed these adverse effects. Compared to the model group, the DFL-treated group showed significantly higher ADG, reduced F/G, and improved ADFI, with effects comparable to those of the JQCS group. This improvement is likely directly related to DFL's anticoccidial activity—by reducing oocyst invasion and mitigating cecal lesions, DFL alleviated the impact of infection on the chicks' digestive and absorptive functions, thereby enhancing nutrient utilization. This aligns with the findings of Yuan et al., (33) who reported that ponazuril improved nutrient absorption by reducing cecal damage through direct anticoccidial action. Moreover, DFL may also enhance growth performance by modulating the intestinal microenvironment or metabolic pathways. For instance, in the study by Yang et al. (11) the Changqing compound improved the intestinal microenvironment by regulating gut microbiota, thereby mitigating the negative impact of *E. tenella* on production performance. These results indicate that DFL not only controls parasitic infection but also significantly improves the economic traits of infected chicks, providing valuable insights for its application in the poultry industry.

The cecum is the primary target of *E. tenella* invasion, often resulting in severe tissue damage following infection. Histopathological analysis revealed that the model group exhibited typical pathological features, including glandular structural disruption, crypt destruction, inflammatory cell infiltration, and widespread invasion by coccidial gametocytes, consistent with the known pathology of *E. tenella* infection in chicks. In contrast, DFL treatment significantly alleviated cecal tissue damage, with largely intact glandular structures and reduced inflammatory cell infiltration. Transmission electron microscopy further confirmed that epithelial villi in the model group were detached and vacuolation was pronounced, whereas in the DFL group, the epithelial villi remained intact and vacuolation was markedly reduced. Tight junction proteins are critical components of the intestinal barrier, playing a central role in maintaining intestinal homeostasis by regulating paracellular permeability and contributing to disease progression and therapeutic interventions (34). Key tight junction proteins include Claudin-1, ZO-1, and Occludin. Claudin-1 enhances the sealing capacity of the intestinal barrier; Occludin dynamically regulates barrier stability through phosphorylation-mediated control of permeability; ZO-1 links transmembrane proteins to the cytoskeleton, maintaining structural integrity. These proteins form continuous belt-like structures that prevent the passive diffusion of macromolecules and pathogens (35). In chicks infected with *E. tenella*, the expression of tight junction proteins in the cecum is disrupted (36). In our study, expression levels of ZO-1, Occludin, and Claudin-1 were significantly reduced in the model group, while DFL treatment restored both mRNA and protein expression of these junctional components, indicating that DFL effectively preserved intestinal barrier integrity. These findings suggest that DFL protects intestinal structure from infection-induced damage by directly inhibiting coccidial invasion of the cecal epithelium and enhancing the expression of tight junction proteins, thus providing structural support for the recovery of chick health.

*E. tenella* infection triggers a pronounced inflammatory response, characterized by elevated mRNA and protein expression of inflammatory markers such as TNF-α, IL-1β, and IL-6 (37). Previous studies have shown that coccidial infection activates host immune responses and signaling pathways. For example, *E. tenella* may activate the NLRP3 inflammasome, promoting IL-1β release (38), and its sporozoites can stimulate host immune cells such as macrophages to upregulate proinflammatory cytokine IL-6 expression (39). In our study, expression of inflammation-related genes in the cecum and levels of inflammatory proteins in serum were significantly increased, indicating severe local and systemic inflammatory responses induced by *E. tenella*. DFL treatment significantly reduced the expression of these inflammatory mediators, demonstrating strong anti-inflammatory effects. Mechanistically, DFL likely exerts its anti-inflammatory effects by inhibiting the activation of NF-κB and MAPK signaling pathways. Specifically, mRNA levels of *NF-*κ*B, TLR-4*, and *P38* were significantly elevated in the model group, while DFL treatment downregulated these genes and upregulated the expression of the anti-inflammatory cytokine *TGF-*β*1*. Serum ELISA further confirmed that DFL reduced the protein levels of TNF-α, IL-6, IL-8, and P38. The study of Yazdanabadi et al. (40) showed that supplementation with arginine also improved the expression of inflammatory factors such as IL-1β, IL-2, IL-6, TNF-α, IFN-γ. Previous research has shown that *E. tenella* infection alters the gut microbiota of chicks, characterized by a reduction in beneficial bacteria and significant enrichment of opportunistic pathogens such as *Escherichia, Enterococcus*, and *Staphylococcus* species (41, 42). Pathogen-associated molecular patterns produced by these bacteria can activate TLR-2 and TLR-4, leading to downstream activation of the NF-κB signaling cascade. NF-κB plays a central role in regulating inflammation by translocating to the nucleus and initiating transcription of proinflammatory cytokines like TNF-α, IL-6, and IL-1β. DFL treatment inhibited this pathway, thereby suppressing the release of inflammatory cytokines. This is consistent with the findings of Meng et al., (43) who reported that both DFL and saikosaponin could attenuate inflammation by inhibiting NF-κB signaling. Additionally, *E. tenella* rhoptry proteins can activate the MAPK signaling pathway via P38, leading to cecal apoptosis and inflammatory damage (44). DFL's inhibition of P38 suggests that it also modulates the MAPK pathway. TGF-β is a key regulator of both innate and adaptive immunity, functioning as a general executor of immune tolerance and a suppressor of inflammation. While excessive TGF-β activity can promote tumor development by suppressing immunity, insufficient activity can result in uncontrolled inflammation and fibrosis (45). In this study, DFL effectively activated TGF-β1, which is crucial for restraining excessive immune and inflammatory responses.

Notably, *in vitro* experiments showed that low-dose DFL (1 μg/ml) significantly inhibited LPS-induced inflammatory gene expression, whereas high doses (20 μg/ml) exhibited pro-inflammatory effects. This indicates a dose-dependent anti-inflammatory effect of DFL, suggesting that dosage optimization is necessary in clinical applications to avoid potential adverse effects. In our previous study, during the drug safety test, doses of 0.1, 0.3, and 0.5 g/kg were administered for 7 days (21). The results indicated that mild swelling of the liver and kidneys occurred only at the five-fold dose of 0.5 g/kg. After a 7-day withdrawal period, the alanine aminotransferase levels decreased compared to the normal group. Therefore, the doses used in this study did not cause any toxic side effects. In summary, DFL alleviates *E. tenella*-induced tissue damage and systemic inflammation through multi-pathway modulation of the inflammatory response, underscoring its potential as a therapeutic agent.

## 5 Conclusion

Through comprehensive *in vivo* and *in vitro* experiments, this study demonstrated that DFL possesses significant therapeutic effects against *E. tenella* infection in chicks. DFL exhibited excellent anticoccidial activity—evidenced by a marked reduction in oocyst shedding and cecal lesion scores (ACI > 170)—and significantly improved growth performance in infected chicks, including increased ADG and reduced F/G. Furthermore, DFL effectively preserved cecal tissue structure, restored the expression of tight junction proteins, and significantly alleviated both local and systemic inflammatory responses by inhibiting the NF-κB and MAPK signaling pathways. These multifaceted therapeutic effects highlight DFL's strong potential in controlling coccidial infection, promoting chick health, and enhancing production efficiency. This study provides solid scientific evidence supporting the development of DFL as a novel anticoccidial agent. However, *in vitro* findings indicate that anti-inflammatory effects of DFL are dose-dependent. Future research should focus on further elucidating its precise mechanisms of action to develop safer and more effective anticoccidial therapies, ultimately contributing to the sustainable development of the poultry industry.

## Data Availability

The original contributions presented in the study are included in the article/supplementary material, further inquiries can be directed to the corresponding authors.
